# Advances in Computational Psychometrics

**DOI:** 10.1155/2015/418683

**Published:** 2015-08-04

**Authors:** Pietro Cipresso, Aleksandar Matic, Dimitris Giakoumis, Yuri Ostrovsky

**Affiliations:** ^1^Applied Technology for Neuro-Psychology Lab, IRCCS Istituto Auxologico Italiano, Via Ariosto 13, 20145 Milan, Italy; ^2^Telefonica I&D, Plaça Ernest Lluch I Marti 5, 08019 Barcelona, Spain; ^3^Information Technologies Institute, Centre for Research and Technology Hellas, 6th Km Charilaou-Thermi Road, P.O. Box 361, 57001 Thessaloniki, Greece; ^4^Wenzhou Medical College, Wenzhou, Zhejiang 325035, China

Advances in computational psychometrics and mathematical methods have been gaining a significant role in both medicine and psychology over these past years. The mainstream in psychometrics is moving towards ever greater use of computational and mathematical modeling techniques. Such techniques are critical in the emerging fields of affective and wearable computing, where new biomedical instruments available both in the laboratory and in the field are allowing for deeper understanding of human psychology. These experimental methods offer new opportunities but also new challenges in data interpretation and analysis.

This special issue has two foci, namely, to feature works that (a) advance scientific knowledge in the area of computational psychometrics and (b) explore deep investigated methods, techniques, and instruments for the assessment of cognitive, emotional, and medical (e.g., diagnostic) as well as mental health at the cutting edge of current technology.

There have recently been an increasing number of research initiatives that utilize computational technologies in order to support patients in maintaining or regaining a healthy mental state. Computational psychometrics and related tools have been exploited for assessing, measuring, and defining new methods for an effective and focused psychological intervention.

It is of utmost importance to provide people with higher quality of life and also to shift a part of monitoring tasks from therapists and caregivers to unobtrusive technological systems. Efforts have started with Internet-based self-help therapies, but recently systems make an increasing use of computational psychometrics, including ambient intelligence, pervasive computing, smart phones, and sensor systems. Their common goal is to provide effective solutions for maintaining and improving mental health and related assessment.

This special issue received many articles, accepting for publication five exciting contributions to the field.

P. Cipresso et al. describe a promising approach for managing data from the interaction of two communicating individuals by collecting multiple electrophysiological signals and eye movements with computational methods. D. Cardone et al. introduced a promising thermal infrared imaging-based tool for the computational assessment of human autonomic nervous activity and psychophysiological states in a contactless and noninvasive way. I. A. C. Giglioli et al. presented a systematic review in the novel field of augmented reality for the assessment and treatment of psychological disorders, highlighting 13 selected articles after an initial screening of 784 articles emerging from the scientific databases. Last but not least, two articles in the exciting field of virtual reality are published: C. Wilson and A. Soranzo review some current uses for VR environments when examining visual perception and discuss limitations or questions that can arise; and S. Segkouli et al. define a more robust cognitive prediction model, accurately fitted to human data to be used for more reliable interface evaluation through simulation on the basis of virtual models of users with mild cognitive impairment (MCI).

We hope that this special issue will foster wider discussion for these exciting themes in computational psychometrics.

*Pietro Cipresso* *Pietro Cipresso* *Aleksandar Matic* *Aleksandar Matic* *Dimitris Giakoumis* *Dimitris Giakoumis* *Yuri Ostrovsky* *Yuri Ostrovsky*

